# Efficacy of hypertonic nasal spray with algae in rhinosinusitis

**DOI:** 10.1007/s00405-025-09266-3

**Published:** 2025-02-20

**Authors:** Zeynel Öztürk, Nuray Bayar Muluk, Rahime Koca, Necdet Özçelіk, Elvin Alaskarov, İbrahim Çukurova, Erdem Atalay Çetіnkaya, Özgür Yörük, Cengiz Bal, Arzu Tatar, Nihat Susaman, Nagehan Dilşad Erdoğmuş Küçükcan, Ayten Güner Atayoğlu, Enes Güngör, Burak Mustafa Taş, Cemal Cingi

**Affiliations:** 1 Istanbul Nişantaşı University, Faculty of Medicine, Department of Otorhinolaryngology and Baypark Hospital, Otolaryngology Clinics, Istanbul, Turkey; 2 Faculty of Medicine, Department of Otorhinolaryngology, Kırıkkale University, Kırıkkale, Turkey; 3 Antalya Training and Research Hospital, ENT Clinic, Antalya, Turkey; 4 Esenler Health Application Center, Medipol University, ENT Clinic, İstanbul, Turkey; 5 Izmir Faculty of Medicine, Department of Otorhinolaryngology, University of Health Sciences, Tepecik Training and Research Hospital, Izmir, Turkey; 6 Faculty of Medicine, Department of Otorhinolaryngology, Atatürk University, Erzurum, Turkey; 7 Faculty of Medicine, Department of Biostatistics, Eskişehir Osmangazi University, Eskişehir, Turkey; 8 University of Health Sciences, Elazığ Fethi Sekin City Hospital, ENT Clinic, Elazığ, Turkey; 9 Adana City Training and Research Hospital, ENT Clinic, Adana, Turkey; 10 Atatürk Family Health Center, Küçükçekmece, İstanbul, Turkey; 11 Faculty of Medicine, Department of Otorhinolaryngology, Eskişehir Osmangazi University, Eskişehir, Turkey; 12 Faculty of Medicine, Department of Otorhinolaryngology, Kırıkkale University, Kırıkkale, Turkey; 13 Birlik Mahallesi, Zirvekent 2. EtapSitesi, C-3 blok, No: 6 − 3/43, Çankaya/Ankara, 06610 Turkey

**Keywords:** Algae-containing hypertonic nasal spray, Hypertonic nasal spray, Isotonic saline nasal spray, Rhinosinusitis, Total symptom scores, Quality of life scores (QoL)

## Abstract

**Objectives:**

We aimed to evaluate the effectiveness of nasal irrigation sprays at treating rhinosinusitis by easing congestion in the nose and other symptoms.

**Methods:**

A total of 1700 individuals diagnosed with rhinosinusitis were assigned to the following groups: One group used Sinomarin^®^ hypertonic nasal spray (*n* = 600), another used Sinomarin^®^ Plus Algae ENT hypertonic nasal spray (*n* = 600), and a third used an isotonic saline nasal spray (*n* = 500). Before and after therapy, patients had their symptoms and turbinates examined, overall symptoms scored, and quality of life (QoL) evaluated.

**Results:**

The findings showed that both groups saw a decrease in symptom scores for anterior discharge, postnasal drip, headache, and obstruction with therapy, as well as an improvement in turbinate color and edema (*p* < 0.05). Quality of life (QoL) ratings rose, and total symptom scores fell during therapy. The group that used hypertonic nasal spray with algae had considerably fewer overall symptom scores than the other groups (hypertonic nasal spray and saline). The hypertonic nasal spray group reported substantially fewer symptoms than the saline group. For what concerns quality of life, the saline group had significantly worse scores than groups 1 and 2; groups 1 and 2 were similar to one another.

**Conclusion:**

We found that compared to hypertonic and saline nasal sprays, the algae-containing hypertonic spray reduced total symptom scores more than the other sprays. This indicated that irrigation based on algae-containing hypertonic nasal sprays could be the treatment of choice for the management of symptoms in rhinosinusitis patients.

**Clinical trial number:**

The study was registered to ClinicalTrials.gov Protocol Registration and Results System (PRS) with a Clinical trial number of E-95961207-202.3.02-2955.

## Introduction

Whether or not nasal polyps are present, chronic rhinosinusitis is defined by inflammation of the nasal cavity and paranasal sinuses [1]. Medical treatments have varying degrees of success with this diverse and frequently refractory condition. The disease has a devastating effect on people’s health and well-being, leading to substantial morbidity [2]. When medical treatments for chronic rhinosinusitis fail, functional endoscopic sinus surgery (FESS) may be an option [1, 3, 4]. Patients with chronic rhinosinusitis must continue to use medical therapy, particularly topical treatment, after FESS because inflammatory processes still play a significant role in their condition [5]. For treating persistent rhinosinusitis following FESS, nasal irrigation is an effective and time-tested supplementary strategy [6, 7].

It is still not known how nasal irrigation works. The physiological effects of saline nasal irrigation on nasal mucosa function include removal of antigens, bacterial biofilm, or inflammatory mediators (thus alleviating inflammation), direct cleaning of mucus (mucus is a potential condition for bacteria to multiply; saline dilutes mucus and helps to clear it out), and improved mucociliary function [8]. Use of irrigation, even with saline, improved symptoms and quality of life more than no irrigation for chronic rhinosinusitis, according to a 2007 Cochrane study [9, 10]. Recent research has demonstrated that a number of topical drugs can be more effectively and efficiently administered through nasal irrigation, leading to higher drug concentrations and better results [11, 12].

For a long time, people have relied on saltwater and saline as a nose wash and congestion remedy. Regarding treating rhinosinusitis, including postoperative care, the most recent European Position Paper on Rhinosinusitis and Nasal Polyps (EPOS) from 2020 states that nasal saline irrigations and sprays are advised [13]. In addition, Rosenfeld et al. have shown that nasal washes are essential and beneficial as a first-line treatment due to their anti-inflammatory effects and ability to reduce the unnecessary use of antibiotics [14]. Hypertonic nasal solutions decrease nasal mucosal edema and enhance nasal ciliary clearance frequency, according to in vitro research by Wang et al. [15].

A study conducted in 2010 by Culig et al. demonstrated that compared to isotonic saltwater, hypertonic solutions were more effective in reducing symptoms such as headache, nasal discharge, congestion, and congestion, as well as enhancing the quality of sleep [16]. Rhinosinusitis symptoms were alleviated by the isotonic and hypertonic nasal sprays tested by Hauptman and Ryan [17], although the hypertonic sprays produced more mucociliary clearance. Compared to a control group, individuals with rhinosinusitis who used hypertonic nasal solution daily for six months had less need for antibiotics and fewer symptoms of the condition, according to research by Rabago et al. [18]. Hypertonic solutions improve mucociliary clearance, according to studies by Bachmann et al. and Hauptman et al. [17, 19].

In this study, we compared the efficacy of three different nasal sprays, an algae-containing hypertonic nasal spray, a hypertonic nasal spray, and a saline solution, in alleviating nasal congestion and lowering symptoms in adult patients with rhinosinusitis.

## Materials and methods

In this prospective, multicenter, observational study, we followed the guidelines laid out in the Declaration of Helsinki. Initially, ethical approval was obtained from Istanbul Medipol University, Traditional and Complementary Medicine Applications Ethics Committee, file number 08, on 27 March 2024. The approval application was made to the Ministry of Health General Directorate of Health Services Traditional and Complementary Medicine Applications on 23 July 2024, with application number 249,334,156. The study was registered to ClinicalTrials.gov Protocol Registration and Results System (PRS) with a Clinical trial number of E-95961207-202.3.02-2955. The patients were asked to verbalize their agreement.

### Subjects

From April 2024 to July 2024, 1.700 patients with rhinosinusitis (852 males and 848 females) who met the inclusion criteria were enrolled in the study. Their symptoms were anterior discharge, postnasal drip, headache, and obstruction; and examination findings were anterior discharge, postnasal drip and hyperemia of the nasal mucosa. The participants came from various centers around Turkey and presented acute or chronic rhinosinusitis symptoms such as post-nasal drip, headache, and nasal blockage. An otolaryngologist examined every patient, and they all kept up with their regular treatment for rhinosinusitis [1] while also using the nasal spray prescribed to them i.e. one spray in each nostril, three times daily, for three weeks.

At ENT clinics, patients were divided into three groups based on the attending physician’s order. The first arriving patient was included in group 1, the next arriving patient in group 2, and the next arriving patient in group 3. Subsequent patients were included in groups according to this order, and the spray treatments specified below were administered to the patients:

Group 1: The first group included six hundred (*n* = 600) people and used Sinomarin^®^, a 2.3% NaCl hypertonic seawater solution [20].

Group 2: The second group included six hundred (*n* = 600) people and used Sinomarin^®^ Plus Algae ENT, a 2.3% NaCl hypertonic seawater solution further containing sea algae extracts from Undaria Pinnatifida and Spirulina platensis [21].

Group 3: The third group with five hundred (*n* = 500) participants used Berko-fiz^®^ Nasal Spray, an isotonic saline nasal spray.

### Volunteer eligibility requirements

1. Beginning in April 2024, patients who presented themselves for a regular ENT (Ear, Nose, and Throat) outpatient exam at one of the designated centers and were found to have rhinosinusitis based on their symptoms, medical history, and examination findings.
2. Individuals who consented to participate in the study.
3. All individuals who are 18 years old or older.

### Exclusion criteria for volunteers

1. Individuals suffering from allergic rhinitis.
2. People who suffered from mental illness.
3. Pregnancy.
4. Individuals diagnosed with sinonasal or other types of cancer.
5. Fatal heart attack.
6. People with a history of heart conditions, like high blood pressure.
7. People whose immune systems were suppressed by medication.
8. Individuals who were impaired mentally.

Patients’ total symptom scores, QoL scores, and Otolaryngology examination scores were assessed before and three weeks following therapy.

## Methods

The researchers had everyone who took part in the study fill out a questionnaire assessing the following rhinosinusitis symptoms: anterior discharge, postnasal drip, headache, and obstruction scores. The scale used had a grading from 1 to 5, starting at 1 for very few symptoms and going up to 5 for severe symptoms. Total symptom scores, which ranged from 1 to 20, were also calculated in the same way.

The Quality of Life scale ran from 1 (the worst possible quality) to 10 (the best possible quality).

In addition, the outcomes of an otolaryngological examination were used to rate the patients. Scales of 1 to 3 were used to assess the turbinates’ color (natural, slightly reddish, and severe hyperemia), as well as the degree of edema (none, mild, or substantial).

### The statistical analysis

The analysis was conducted using the statistical software package SPSS for Windows 21.0, developed and owned by IBM (Chicago, Illinois). The Kolmogorov-Smirnov test was used to check if the variables followed a normal distribution. When comparing groups, both parametric and non-parametric tests were employed. For means based on distribution forms, we utilized the Student’s t-test, and for pre- and post-scores, we used the Wilcoxon signed-rank test. Chi-square tests were used to analyze the cross-tables produced for the independent variables. The cross-tables (crosstabs) that were made to compare symptoms (discharge, sneezing, itching, congestion, turbinate color, and turbinate edema) before and after treatment were analyzed using McNemar-Bowker tests.

The Kruskal-Wallis test was utilized to compare symptom ratings and quality of life values among the three groups according to their distribution forms. A post hoc test was then performed for multiple comparisons.

Count (%) was utilized in the data summary for qualitative data, whereas Mean ± SD or Median (Q1; Q3) statistics were employed for quantitative data. Statistical significance was determined when *p* < 0.05.

## Results

### Group 1 (hypertonic nasal spray group)

In group 1, there were 291 (48.5%) males and 309 (51.5%) females (*n* = 600). The mean age of group 1 was 36.66 ± 12.12 years.

#### Symptom scores

There were significant differences between pre-treatment and post-treatment anterior discharge, postnasal drip, headache, and obstruction scores (*p* < 0.001). All of them decreased after treatment.

#### Evaluation of turbinates

There was a significant improvement in the turbinate color and edema scores after treatment compared to pre-treatment values (*p* < 0.001).

### The total symptom and quality of life scores before and after treatment are shown in Table 1

**Table 1:** Total symptom score and QoL scores at pre- and post-medication period

		Pre-medication			Post-medication			p
		Median	Q1	Q3	Median	Q1	Q3	
Group 1 (Hypertonic nasal spray) (*n* = 600)	Total Symptom Score (1–20)	10.0	9.0	13.0	6.0	5.0	9.0	**< 0.001**
	Quality of life score (1–10)	7.0	6.0	8.0	8.0	7.0	8.0	**< 0.001**
Group 2 (Algae-containing hypertonic nasal spray) (*n* = 600)	Total Symptom Score (1–20)	10.50	9.0	13.0	5.0	4.0	8.0	**< 0.001**
	Quality of life score (1–10)	7.0	5.0	7.0	8.0	7.0	8.0	**< 0.001**
Group 3 (Isotonic saline nasal spray) (*n* = 500)	Total Symptom Score (1–20)	10.0	9.0	13.0	10.0	9.0	13.0	0.054
	Quality of life score (1–10)	7.0	5.0	7.0	7.0	6.0	8.0	**< 0.001**

The median total symptom scores decreased after treatment than pre-treatment scores (*p* < 0.001). The median quality of life scores increased after treatment than pre-treatment scores (*p* < 0.001).

### Group 2 (Algae-containing hypertonic nasal spray group)

In group 2, there were 309 (51.5%) males and 291 (48.5%) females (*n* = 600). The mean age of group 1 was 36.71 ± 12.40 years.

#### Symptom scores

There were significant differences between pre-treatment and post-treatment anterior discharge, postnasal drip, headache, and obstruction scores (*p* < 0.001). All of them decreased after treatment.

#### Evaluation of turbinates

There was a significant improvement in the turbinate color and edema scores after treatment compared to pre-treatment values (*p* < 0.001).

### The total symptom and quality of life scores before and after treatment are shown in Table 1

The median total symptom scores decreased after treatment than pre-treatment scores (*p* < 0.001). The median quality of life scores increased after treatment than pre-treatment scores (*p* < 0.001).

### Group 3 (Isotonic saline nasal spray group)

In group 3, there were 252 (50.4%) males and 248 (49.6%) females (*n* = 500). The mean age of group 3 was 36.75 ± 11.75 years.

#### Symptom scores

There were no significant differences between pre-treatment and post-treatment anterior discharge (*p* = 0.135), postnasal drip (*p* = 0.147), headache (*p* = 0.051), and obstruction scores (*p* = 0.062).

#### Evaluation of turbinates

There was a significant improvement in the turbinate color and edema scores after treatment compared to pre-treatment values (*p* < 0.001).

### The total symptom and quality of life scores before and after treatment are shown in Table 1

There were no significant differences in the median total symptom scores before and after treatment (*p* = 0.054). The median quality of life scores before and after treatment were found to be different (*p* < 0.001). The median quality of life scores increased after treatment.

### The total symptom score and quality of life score analysis between 3 groups

At the pre-treatment period, there were no significant differences between the 3 groups’ total symptom scores (*p* = 0.663) and quality of life scores (*p* = 0.173).

During the post-treatment period, there were statistically significant differences between the total symptom scores of the 3 groups (*p* < 0.001). Total symptom scores of group 2 (Median 5.0) were significantly lower than those in group 1 (Median 6.0) and 3 (Median 10.0) (*p* < 0.001). The total symptom scores of group 1 (Median 6.0) were significantly lower than those in group 3 (Median 10.0) (*p* < 0.001).

During the post-treatment period, there were statistically significant differences between the quality of life scores of the 3 groups (*p* < 0.001). Quality of life scores of group 1 (Median 8.0) (*p* < 0.001) and group 2 (Median 8.0) (*p* < 0.001) were significantly higher than those in group 3 (Median 7.0). There were no significant differences between group 1 (Median 8.0) and 2 quality of life scores (Median 8.0) (*p* = 0.751).

## Discussion

Nasal irrigation is an efficient technique resulting in the mechanical wash of mucous and any infectious, inflammatory or other agents (e.g. allergens, pollutants etc.) embedded in it. Nasal washes additionally increase hydration in the deeper aqueous layer, thereby enhancing the underlying ciliary beat frequency [22]. Although both isotonic and hypertonic solutions are efficacious, hypertonic solutions further offer an osmotically-induced decongestive effect in nasal cavities; these properties are particularly helpful in treating sinonasal illnesses [22–24].

A series of studies has shown that nasal irrigation is especially beneficial in the case of upper respiratory tract infections. For example, Ramalingam and co-workers showed that hypertonic nasal irrigation and gargling was efficacious in reducing viral load, duration of illness and disease transmission in patients with common cold, including patients infected by SARS-CoV-2 (25–26). In another study, using a hypertonic alkaline nasal irrigation solution, Yilmaz et al. showed that the nasopharyngeal viral load in COVID-19 patients decreased significantly [27]. Finally, utilizing 79 COVID-19 patients in their outpatient trial, Baxter and co-workers proved the practicability of instructing and assisting patients using nasal irrigation devices [28].

In the recent years, and boosted by the COVID-19 pandemic, irrigation solutions comprising additional algal ingredients have also been developed and tested in a series of preclinical and clinical trials [29]. This effort was triggered by preclinical results showing that sulphated polysaccharides present in algal extracts impede SARS-CoV-2 binding to host tissues therefore making it unable to infect [30]. Among different types of molecules tested, brown algae-derived fucoidan sulphated polysaccharides (e.g. fucoidans derived from Undaria pinnatifida) were among the most active [31, 32]. In addition, their activity extended to several enveloped respiratory viruses other than SARS-CoV-2 making them good candidates for inclusion in solutions used for treatment of all viral upper respiratory tract diseases [30]. Molecules isolated from blue/green algae such as Spirulina, were also shown to possess similar viral entry inhibitory effects [33]. In addition to these physical properties, sulphated polysaccharides have also been shown to retain moisture, offering hydrating effects [34]. This property may also be relevant in case of respiratory tract infections since recent studies suggest that targeted hydration of the nose, larynx and trachea using irrigation solutions has therapeutic benefits [35].

In this clinical study, we have looked at how three different nasal sprays, one with a 2.3% NaCl hypertonic seawater solution (Group 1), one with a 2.3% NaCl hypertonic solution further containing algae derived from Undaria pinnatifida and Spirulina platensis (Group 2), and one with an isotonic saline solution (Group 3) impacted rhinosinusitis. Our results showed that after therapy, turbinate color and edema improved, and headache severity, postnasal drip, anterior discharge, and obstruction ratings all decreased in the algae-containing hypertonic nasal spray. Quality of life (QoL) ratings rose, and total symptom scores fell during therapy. Treatment resulted in no statistically significant changes in symptom scores or total symptom scores in the saline group, however turbinate color venous edema values did improve. Quality of life scores improved following therapy.

The group that used the algae-containing hypertonic nasal spray had considerably fewer overall symptom scores than the two other groups (hypertonic nasal spray and saline). The hypertonic nasal spray group reported substantially fewer symptoms overall compared to the saline group. Compared to the saline group, the hypertonic nasal spray groups 1 and 2 had much better quality of life scores. Groups 1 and 2 were similar to one another for what concerns quality of life scores. These results suggest that both hypertonic nasal sprays (with or without algae) offer therapeutic activity and are superior to isotonic solutions in patients with rhinosinusitis. Our results also suggest that the addition of algae results in overall better symptom management versus that offer by the hypertonic solution that does not contain algae. This effect may be explained by the physicochemical properties of the algae, namely their ability to hydrate the nasal passages via moisture retention and possible physical barrier effects by the contained sulphated polysaccharides, as mentioned in detail above.

Overall, our observations and clinical experience suggest that hypertonic solutions with or without algae are highly effective in treating symptoms of rhinosinusitis. These results are in agreement with clinical trials conducted with the same 2.3% NaCl hypertonic solution in patients with ENT disorders [24] or trials using the same or similar algae-based 2.3% hypertonic seawater solutions. These included trials in allergic rhinitis [36], in COVID-19 patients (37–38) or patients at the post-operative setting (39–40).

## Conclusion

In our trial, hypertonic seawater solutions were highly effective and superior to isotonic solutions in managing symptoms of patients with rhinosinusitis. An algae-based hypertonic seawater solution comprising extracts from Undaria pinnatifida and Spirulina platensis showed the best therapeutic activity among the nasal irrigation solutions tested. These results indicate that algae-based hypertonic solutions may be the irrigation treatments of choice in the management of rhinosinusitis. Therefore, hypertonic seawater solutions can be considered for concomittant nasal medication and can be recommended besides other treatments of rhinosinusitis.

## Data Availability

All data for this study is presented in this paper.
